# Investigation of Potential In Vitro Anticancer and Antimicrobial Activities of *Balanites aegyptiaca* (L.) Delile Fruit Extract and Its Phytochemical Components

**DOI:** 10.3390/plants11192621

**Published:** 2022-10-05

**Authors:** Omer H. M. Ibrahim, Adel D. Al-Qurashi, Khalid A. Asiry, Magdi A. A. Mousa, Nabil A. Alhakamy, Kamal A. M. Abo-Elyousr

**Affiliations:** 1 Department of Arid Land Agriculture, Faculty of Meteorology, Environment and Arid Land Agriculture, King Abdulaziz University, Jeddah 21589, Saudi Arabia; 2 Department of Pharmaceutics, Faculty of Pharmacy, King Abdulaziz University, Jeddah 21589, Saudi Arabia; 3 Center of Excellence for Drug Research and Pharmaceutical Industries, King Abdulaziz University, Jeddah 21589, Saudi Arabia; 4 Mohamed Saeed Tamer Chair for Pharmaceutical Industries, Faculty of Pharmacy, King Abdulaziz University, Jeddah 21589, Saudi Arabia

**Keywords:** antibacterial, anticancer, antifungal, apoptosis, *Balanites aegyptiaca*, cell cycle arrest, necrosis

## Abstract

The therapeutic importance of *Balanites aegyptiaca* in folk medicine for the treatment of several common human diseases has led researchers to conduct phytochemical and pharmacological studies on extracts from various parts of the plant. In the current study, the phytochemical composition of the *B. aegyptiaca* methanolic fruit extract was characterized, and its antimicrobial activity was evaluated together with the cytotoxic activity against MCF-7, PC-3, and Caco-2, compared with normal Vero cells. Further, its effects on cell cycle arrest, apoptosis induction and expression of apoptosis-related genes were assessed. The phytochemical screening revealed the presence of fatty acids and their esters in addition to phytosterols, steroid derivatives, and bioflavonoid glycosides with oleic and palmitic acids being the prevalent components (24.12 and 21.56%, respectively). The results showed considerable cytotoxic activity of the extract against the three cancer cell lines (MCF-7, PC-3, and Caco-2) with a selectivity index ranging from 5.07 to 6.52. This effect was further confirmed with the accompanied increased total apoptosis of treated PC-3 cells (19.22% of the total number of cells) compared to the control cells (0.64% of the total number of cells) with cell cycle arrest at G1 phase and the increased transcription of pro-apoptotic genes including *P53* (3.69) and *BAX* (3.33) expressed as fold change (2^ ΔΔCT). The calculated minimum inhibitory concentration (MIC) was similar (62.5 µg/mL) against the three tested bacterial strains (*Acinetobacter johnsonii*, *Serratia marcescens* and *Agrobacterium tumefaciens*), while it was higher than 1000 µg/mL for the fungal species (*Rhizoctonia solani*, *Penicillium italicum*, and *Fusarium oxysporium*). Our findings suggest a promising anticancer activity for *B. aegyptiaca*, which paves the way for more detailed future studies.

## 1. Introduction

Some plant species are described as neglected and underused despite their known horticultural and nutritional values that supported their adaptation to low-input agriculture [1,2]. One of these species is *Balanites aegyptiaca* (L.) Delile, which belongs to family *Zygophyllaceae* and is known as heglig or desert date, referring to its edible fruits [3]. Some recent reports confirmed its suitability for the restoration of forest landscapes and degraded lands [4] and its cultivation as an oil crop in arid lands for large-scale sustainable industrial biodiesel production [5], in addition to being occasionally planted for ornamental purposes [6]. Further considerable importance for its timber in furniture and charcoal production has been stated [7]. The edible fruits of *B. aegyptiaca* have been reported in folklore medicine for treating whooping cough, leukoderma, and other skin diseases [8,9], oral hypoglycemic [9], antidiabetic and jaundice therapy [10]. Uses for leaves, roots, and bark have also been reported [7]. Ethnobotanical uses of this plant species have recently led to extensive phytochemical and pharmacological studies on the therapeutic efficacy of *B. aegyptiaca* against many human diseases. 

The nutritional and phytochemical composition of different plant parts together with their biological activities have been reviewed by several authors [7,8,11,12,13]. A variety of secondary metabolites have been isolated from various plant parts, including phenols, alkaloids, steroids, and saponins. Literature data show considerable variation in bioactive compounds and biological activities of *B. aegyptiaca* of various origins based on geographical and environmental conditions [14]. Differences in phytochemical composition, including oil, protein, and some minerals, together with the antioxidant activity of fruits collected from different trees of *B. aegyptiaca,* were reported by several authors such as Abdel-Farid and El-Sayed [15] and Abdelaziz et al. [4]. According to several studies, *B. aegyptiaca* seeds contain approximately 38–57% fats with palmitic and oleic acids being the prevalent fatty acids estimated to be around 40% of seed oil [1,3,7,8,16,17]. Other reports showed that the oleic content in the fruits ranged from 33.5% to 69.6% and palmitic from 15.0–17.9% [18] Phytosterols including *β*-Sitosterol and stigmasterol were also previously reported in fruit extracts [3]. 

A wide range of biological activity has been confirmed for *B. aegyptiaca* extracts including antioxidant, anticancer, antidiabetic, anti-inflammatory, antimicrobial, hepatoprotective and molluscicidal activities [7]. Recent studies have confirmed the medicinal use of different extracts of *B. aegyptiaca* fruits in the treatment of hyperactive gut disorders [19], diabetes mellitus [20], hyperglycemia [21], and dermatophytes [22]. The antibacterial activity of *B. aegyptiaca* fruit extract has been reported by several previous studies against a number of fungal and bacterial species [3,22,23,24,25,26,27]. These effects are ascribed to phytochemicals detected in the extract including palmitic acid [28,29] and hexadecanoic acid, 2,3-dihydroxypropyl ester. Cytotoxic effects of *B. aegyptiaca* fruit extract were revealed by Al-Malki [30] against Hep-2, MCF-7, HL-60, and HCV29T cells. The oil and the comprised fatty acids exhibited anticancer activity against lung, liver, and brain human carcinoma cell lines [3,31,32]. Examples of promising anticancer fatty acids include hexadecanoic acid (palmitic acid) [33], palmitic acid [28,34], and oleic acid [35]. Sterols and steroidal derivatives present in the extract have also proved a potential anticancer [36,37] including ethyl isoallocholate against A549 lung cancer cells [38]. Several studies elucidated therapeutic signaling pathways of *B. aegyptiaca* fruit extract components against cancer cells. These effects could be exerted through the elevation in ROS (reactive oxygen species) production leading to the activation of the apoptotic pathway through the caspase-independent pathway [39]. Another pathway is the caspase-dependent mitochondrial apoptosis, which together with the caspase-independent pathway can be triggered upon activation of the intrinsic cell death pathway [40]. In line with that, Issa et al. [41] recorded antiproliferative activity of *B. aegyptiaca* extract accompanied by a significant reduction in lipid peroxidation, but elevated activities of superoxide dismutase and catalase and expression of P53 gene expression.

In the current work, the main objective is to investigate the phytochemical composition of *B. aegyptiaca* methanolic fruit extract and its antimicrobial activity. Further, the potential proapoptotic activity of the extract is assessed against PC-3, MCF-7, and Caco-2 cell lines.

## 2. Materials and Methods

### 2.1. Chemicals and Reagents

The microbial media were purchased from HiMedia company, India including potato dextrose agar (PDA) and NSA (nutrient-sucrose agar) media. Standard chemicals and GC-grade solvents were purchased from Sigma-Aldrich Chemie GmbH (Taufkirchen, Germany). All chemicals used for the cytotoxicity assay were purchased from Bio Basic Inc. (Markham, ON, Canada), including RPMI-1640 medium, MTT (3-(4,5-dimethylthiazol-2-yl)-2,5-diphenyltetrazolium bromide), fetal bovine serum, DMSO (Dimethyl sulfoxide), and PBS (Phosphate-Buffered Saline). RNase and propidium iodide were from Abcam (Boston, MA, USA), FITC Annexin V with PI from BioVision, (Milpitas, CA, USA) and RT-PCR kits and oligonucleotide primers from Bio-Rad (Hercules, CA, USA).

### 2.2. Preparation of B. aegyptiaca Fruit Extract 

A desert date tree (*B. aegyptiaca*) naturally growing in Aswan governorate in Egypt’s southern desert, downstream of Wadi Allaqi was used as a source of fruit samples. The fruits collected were delivered to the Department of Ornamental Plants and Landscape Gardening, Assiut University, Egypt, where senior staff members verified their authenticity and the voucher specimen was deposited at the department under number ASTU-3523. The fruits were rinsed under running water, dried in shade, grinded, and utilized to prepare the methanolic extract. Under constant shaking, 100 g of fruit powder was thoroughly mixed in 100 mL of methanol: water (8:2 *v*/*v*) for 3 days. The same procedures were conducted twice more after filtering the macerate and collecting the filtrate. The mixed filtrates of the three times were combined and subjected to concentration under reduced pressure at 50 °C using Hidolph VV2000 rotary evaporator and then freeze-dried at 1.5·10^−4^ mbar for 48 h using Telstar-LyoQuest plus-55 lyophilizer. The yield of dried extract was estimated (31.1 g corresponding to 31.1% of the dried fruit powder) and stored in dark vials at −20 °C until used for further analyses.

### 2.3. Gas Chromatography–Mass Spectrometry (GC–MS) Analysis

Trace GC-TSQ mass spectrometer (Thermo Scientific, Austin, TX, USA) was employed to identify the phytochemical components of *B. aegyptiaca* fruit extract. The GC-MS was coupled with TG-5MS capillary column (30 m × 0.25 mm × 0.25 µm film thickness). The temperature program was set up so that the oven temperature was 50 °C and then increased by 5 °C /min to 250 °C and held for 2 min and further increased by 30 °C /min to the final temperature (300 °C) and held for 2 min. The injector and the MS transfer line were set to temperatures of 270 and 260 °C, respectively. The carrier gas was helium at a 1 mL/min flow rate. Injection of samples was done in the GC split mode using Autosampler ASI300 with a solvent delay of 4 min. Collection of EI mass spectra was done at 70 eV ionization voltages over the range of *m*/*z* 50-650 in full scan mode and ion source temperature of 200 °C. The detected components were identified by comparing their mass spectra with those of WILEY 09 and NIST 14 databases.

### 2.4. Antibacterial Activity

The agar diffusion method described by Brulez and Zeller [42] and Abo-Elyousr et al. [43] was employed to test the antibacterial activity of the *B. aegyptiaca* fruit extract against three bacterial strains obtained from the strains deposited at Department Plant Pathology, Assiut University, Egypt (*Agrobacterium tumefaciens* 614, *Serratia marcescens* 2039, and *Acinetobacter johnsonii* 6005). The suspension of tested bacterial species was spread over NSA (nutrient-sucrose agar) medium and kept until dry before applying the treatments with different concentrations of the extract (15.6, 31.3, 62.5, 125, 250, 500, and 1000 µg/mL) in a 9 mm punch. Amoxicillin (62.5 µg/mL) was used as a positive control and all treatments were replicated four times. All treated plates were incubated at 27 °C for 2 days, after which the zones of inhibition were recorded for the control (A) and the treatments (B). The percentage of growth inhibition was estimated as per Equation (1) and the minimum inhibitory concentration (MIC) was assessed as the lowest extract concentration that suppresses the bacterial growth [44].

(1) $$\mathrm{Growth}\;\mathrm{inhibition}=\left(\frac{A-B}{A}\right)\text{×}100$$

### 2.5. Antifungal Activity

The in vitro activity of *B. aegyptiaca* fruit extract was assessed against three fungal species obtained from the strains deposited at Department Plant Pathology, Assiut University, Egypt (*Rhizoctonia solani* 301, *Penicillium italicum* 309, and *Fusarium oxysporium* 389) according to the method previously reported by Abdel-Hafez et al. [45]. Different concentrations of the extract (15.6, 31.3, 62.5, 125, 250, 500, and 1000 µg/mL) and hymexazol at 1000 µg/mL (positive control) were prepared in PDA (Potato Dextrose Agar) medium and poured into Petri plates. The inoculation with fungal growth was done by transferring a 2 mm fungal plug of the correspondent fungus into the center of each plate, after which the cultures were incubated at 28 °C for 10 days. The diameters of fungal colonies were measured in the treated plates (B) when the untreated control plates (A) were fully covered by mycelia growth of the tested fungus. The percentage of growth inhibition was estimated as per Equation (1).

### 2.6. Cytotoxicity Test In Vitro

#### 2.6.1. Cell Cultures

The cell lines employed in the current study were obtained from the American Type Culture Collection (ATCC, Manassas, VA, USA) and included three human cancer cell lines, i.e., the prostate (PC-3, accession number: ATCC CRL-1435), breast (MCF-7, Accession number: ATCC HTB-22), and colorectal adenocarcinoma (Caco-2, accession number: ATCC ATB-37), together with the normal Vero cell line (accession number: ATCC CCL-81). RPMI medium was used to grow the cells with the addition of 10% fetal bovine serum, 100 units/mL penicillin G and 100 mg/mL streptomycin sulfate. Cultured cells were incubated at 37 °C in a CO_2_ incubator and were then harvested with the help of trypsin at 0.25% and EDTA-2Na at 0.025% in PBS. 

#### 2.6.2. MTT Assay

In vitro cytotoxicity of *B. aegyptiaca* extract was studied using MTT assay as per the method described in detail in our previously published work [46,47]. Concisely, cultured cells were harvested and plated in 96-well plates containing a final volume of 100 µL/well (1 × 10^5^ cells/mL) and incubated for 24 h at 37 °C in a CO_2_ incubator. The developed cell monolayer was washed twice with a fresh growth medium. Different concentrations of the extract (31.25, 62.5, 125, 250, 500 and 1000 µg/mL) were prepared using a maintenance medium (RPMI-1640 medium augmented with fetal bovine serum at 2%). The cells were treated with different extract concentrations along with doxorubicin as the positive control and incubated for 48 h. Then the medium was removed and MTT solution (5 mg/mL) was added to each well at 20 mL/well and incubated for 4 h under dark conditions. After that, the MTT was decanted and DMSO (dimethyl sulfoxide) was added at 200 µL/well and incubated again for 30 min. The optical densities of the plated cells were recorded at 560 nm wavelength using an ELISA (enzyme-linked immunosorbent assay) reader. Calculation of the cytotoxicity percentage was done according to Equation (2).

(2) $$\mathrm{Cytotoxicity}\;\mathrm{percentage}=\left(\frac{A_{560}\;\mathrm{control}\;-{\;A}_{560}\;\mathrm{sample}}{A_{560}\;\mathrm{control}}\right)\text{×}100$$

The IC_50_ was calculated for the extract and doxorubicin against each cell line, and the selectivity index (SI) was calculated according to Equation (3).

(3) $$\mathrm{SI}=\left(\frac{{\mathrm{IC}}_{50}\;\mathrm{normal}\;\mathrm{cells}}{{\mathrm{IC}}_{50}\;\mathrm{cancer}\;\mathrm{cells}}\right)$$

### 2.7. Cell Cycle Arrest Assessment 

According to the procedure previously outlined by Alqahtani et al. [48] and Nasr et al. [49], the cell cycle distribution was evaluated. PC-3 cells were cultured for 24 h after being treated with *B. aegyptiaca* extract at the previously estimated IC_50_ (92 µg/mL). The cells were then removed, thrice rinsed in cold PBS, fixed in cold ethanol (70%), and kept at 4 °C for four hours. PBS was used to rehydrate the fixed cells followed by the addition of RNase A (100 g/mL) and propidium iodide (100 g/mL, Abcam, Boston, MA, USA) for DNA staining. Using BD FACSCalibur flow cytometer with CellQuest software (BD Biosciences, San Diego, CA, USA), the DNA content was calculated after 30 min of incubation. Propidium iodide fluorescence intensity was collected on FL_2_ of a flow cytometer and 488 nm laser excitation.

### 2.8. Assessment of Apoptotic vs. Necrotic Cells

Assessment of apoptotic vs. necrotic PC-3 cells was conducted using FITC Annexin V with PI (BioVision, Milpitas, CA, USA) following the manufacturer’s instructions. PC-3 cells were incubated for 24 h in 6-well plates at 4 × 10^5^ cells/well, and then subjected to the *B. aegyptiaca* extract treatment (92 µg/ mL). The cells were collected and resuspended in Annexin V-binding buffer (100 µL) and then stained using 5 μL of Annexin V-FITC and 5 μL of propidium iodide dyes for 15 min in dark. Apoptotic vs. necrotic cells were then estimated using BD FACSCalibur flow cytometer with CellQuest software (BD Biosciences, San Diego, CA, USA).

### 2.9. Assessment of Apoptosis-Related Gene Expression

The oligonucleotide primers used in the real-time qRT-PCR analysis of the targeted genes included BCL2 (F: 5′-AAG CCG GCG ACGACT TCT-3′, R: 5′-GGT GCC GGT TCA GGTACTCA-3′), BAX (F: 5′-ATGGACGGGTCCGGGGAG-3′, R: 5′-ATCCAGCCCAACAGCCGC-3′), and P53 (F: 5′-ATGTTTTGCCAACTGGCCAAG -3′, R: 5′-TGAGCAGCGCTCATGGTG-3′). The housekeeping gene used to normalize and compare expression was β-actin (F: 5′-ATCGTGGGGCGCCCCAGGCAC-3′, R: 5′-CTCCTTAATGTCACGCACGATTTC-3′). PC-3 cells (4 × 10^5^ cells/mL) plated in a 6-well plate were exposed to B. aegyptiaca extract at 92 μg/mL for 24 h. Total RNA from PC-3 cells was isolated using a TRIzol reagent and the complementary DNA (cDNA) was synthesized from 1 μg of extracted RNA with BioRad syber green PCR MMX kit, as per the manufacturer’s instructions. Quantification of gene expression was done using Rotorgene RT- PCR system (Corbett Research, Sydney, Australia) as described previously by Nasr et al. [49]. The thermal profile applied started with a 5-min incubation at 95 °C for 5 min followed by 45 PCR cycles of 10 s at 95 °C, 30 s at 55 °C and 20 s at 72 °C. The mRNA expression was expressed as fold change determined using 2^ΔΔCt^ method between the non-treated and treated cells. The data were generated by Rotor-Gene 6000 Series Software 1.7 (Build 87). 

### 2.10. Statistical Analysis 

One-way ANOVA was applied to detect differences between more than two groups, while the unpaired t-test was used to evaluate differences between the two groups. The comparison of means was done using LSD test at a P value of 0.05. All data were presented as mean ± SD derived from at least three replicates. The analysis was performed using Statistix software (ver. 8.1, Analytical Software, Tallahassee, FL, USA). 

## 3. Results

### 3.1. GC-MS Analysis

**Figure 1:**
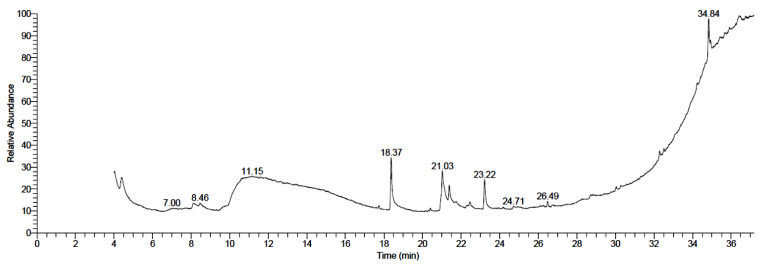
GC–MS chromatogram of *Balanites aegyptiaca* fruit extract.

As represented in Table 1 and Figure 1, the GC–MS analysis for the methanolic fruit extract of *B. aegyptiaca* revealed eight compounds, which are classified as fatty acids and fatty acid esters, phytosterols, and steroid derivatives and isoflavonoid glycosides. Both palmitic and oleic acids were the predominant components with almost 46% of the total peak area. These were followed by Stigmast-5-en-3-ol, (3á)- and 6,9-Octadecadiynoic acid, methyl ester detected in the extract in moderate amounts (15.75 and 12.78%, respectively). Four other components were present in lower content including ethyl iso-allocholate (8.27%) Octadecanoic acid, ethyl ester (7.58%), Octadecanoic acid, 2,3-dihydroxypropyl ester (7.12%) and Flavone-4’-OH,5-OH,7-di-O-glucoside (3%). 

### 3.2. Antibacterial Activity

**Figure 2:**
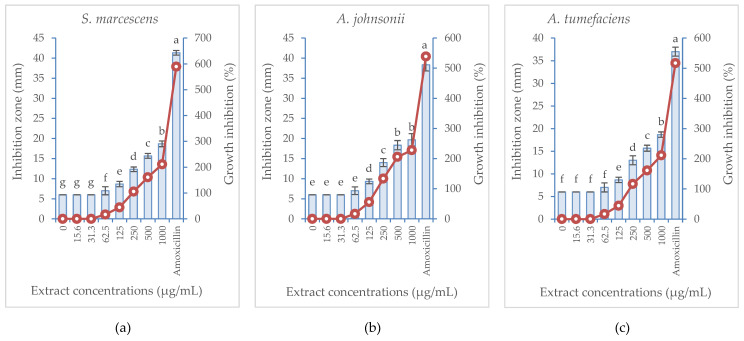
Inhibition zone (vertical bars on primary axis) and growth inhibition percentage (line on secondary axis) of three bacterial species in response to the treatment with *B. aegyptiaca* methanolic fruit extract at various concentrations compared with amoxicillin at 62.5 µg/mL: (**a**) *Seratia marcescens*, (**b**) *Acinetobacter johnsonii*, (**c**) *Agrobacterium tumefaciens*. Values are represented as the mean (*n* = 4) ± SD indicated by the vertical bars.

**Figure 3:**
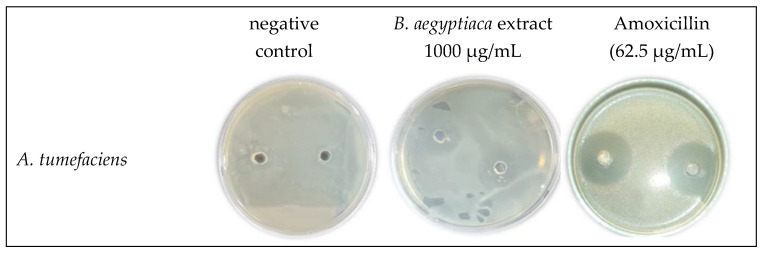

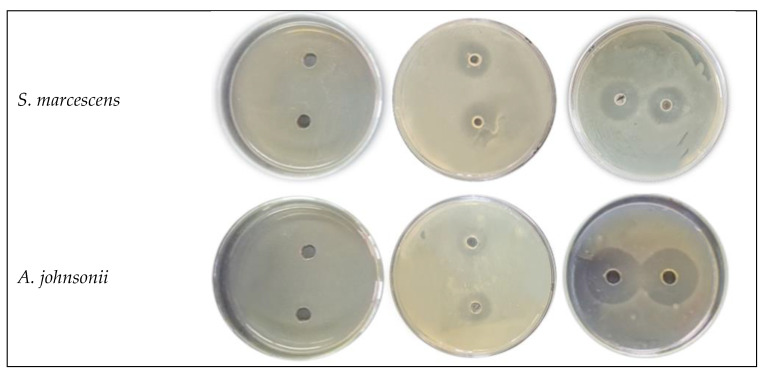
Growth inhibition of three bacterial species: *Seratia marcescens*, *Acinetobacter johnsonii*, and *Agrobacterium tumefaciens*, on NSA medium in response to the treatment with *B. aegyptiaca* methanolic fruit extract at the highest concentration (1000 µg/mL) compared with the negative control and amoxicillin at 62.5 µg/mL.

The potential activity of *B. aegyptiaca* fruit extract was tested against three different bacterial strains, two of which are Gram-positive (*A. johnsonii* and *S. marcescens*) and one is Gram-negative (*A. tumefaciens*), and the results are illustrated in Figure 2 and Figure 3. The treatment with *B. aegyptiaca* extract exerted similar effect against both the bioagent bacterium *S. marcescens* and the plant pathogenic bacterium *A. tumefaciens*, yet it was apparently more pronounced against the human pathogenic bacterium *A. johnsonii*. The lowest extract concentration that suppressed the bacterial growth (MIC) was similar for the three tested strains (62.5 µg/mL). The extract treatment provoked a concentration-dependent growth inhibition % of the three tested bacterial strains. The maximum values recorded for growth inhibition of *S. marcescens*, *A. johnsonii* and *A. tumefaciens* were 211.11, 227.78, and 211.11%, respectively, in response to the highest concentration (1000 µg/mL) of the extract. Meanwhile, the growth inhibition % induced by the positive control treatment (Amoxicillin at 62.5 µg/mL) reached 588.89, 538.89, and 516.67% for the three bacterial strains, respectively.

### 3.3. Antifungal Activity

**Figure 4:**
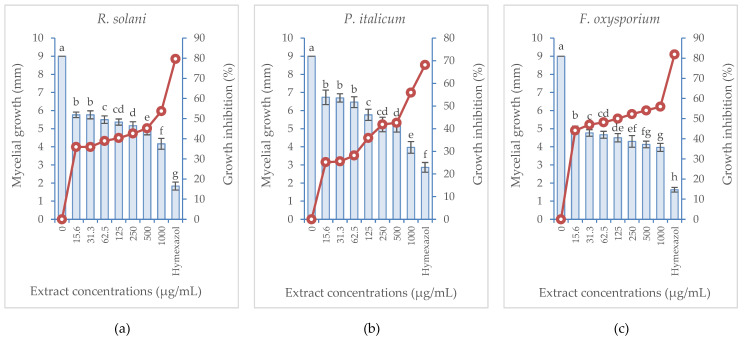
Mycelial growth (vertical bars on primary axis) and growth inhibition percentage (line on secondary axis) of three fungal species in response to the treatment with *B. aegyptiaca* methanolic fruit extract compared with hymexazol at 1000 µg/mL: (**a**) *Rhizoctonia solani*, (**b**) *Penicillium italicum*, (**c**) *Fusarium oxysporium*. Values are represented as the mean (*n* = 4) ± SD indicated by the vertical bars.

**Figure 5:**
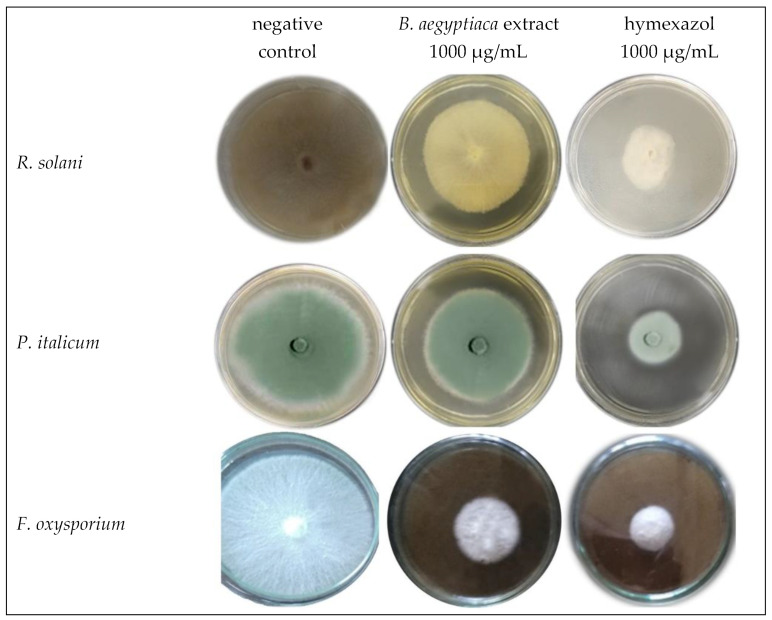
Growth inhibition of three fungal species: *Rhizoctonia solani*, *Penicillium italicum,* and *Fusarium oxysporium*, on PDA medium in response to the treatment with *B. aegyptiaca* methanolic fruit extract at the highest concentration (1000 µg/mL) compared with the negative control and hymexazol at 1000 µg/mL.

*B. aegyptiaca* fruit extract showed evidenced antifungal activity against three different fungi species (*R. solani*, *P. italicum*, and *F. oxysporium*) as displayed in Figure 4 and Figure 5. The fungal growth exhibited significant inhibition as the extract concentration was increased recording the lowest mycelial growth (4.17, 3.97, and 3.97 mm) and hence the highest growth inhibition percentage (53.70, 55.93, and 55.93%) against *R. solani*, * P. italicum,* and *F. oxysporium*, respectively, in response to the treatment with the highest extract concentration (1000 µg/mL). Employing hymexazol (the positive control) at the same concentration (1000 µg/mL) generated significantly higher growth inhibition percentages (79.63, 68.15 and 81.85, respectively) against the three fungi species. It is also noticed that *F. oxysporium* was the most susceptible fungal species to the treatment of either the extract or hymexazol. 

### 3.4. In Vitro Cytotoxic Activity

**Figure 6:**
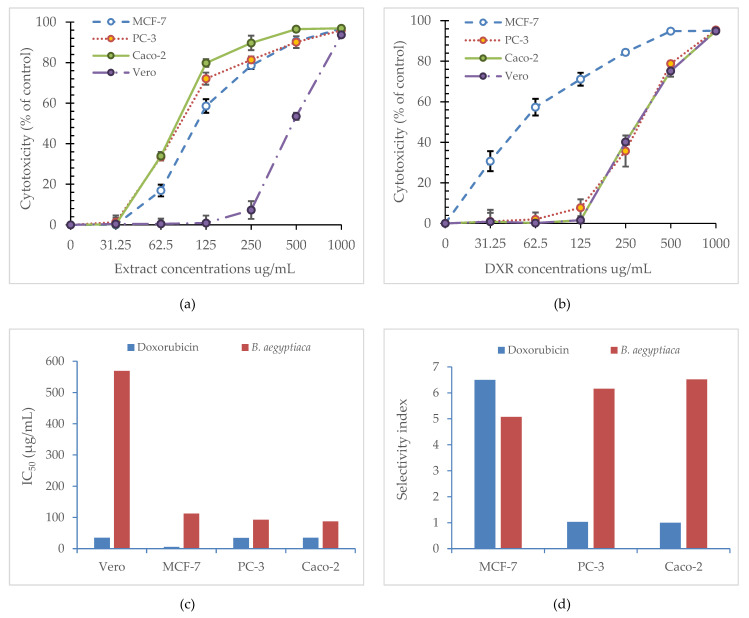
Cytotoxicity response of *B. aegyptiaca* methanolic fruit extract compared with doxorubicin on MCF-7, PC-3, Caco-2, and Vero cells: (**a**) cytotoxic effect of the extract, values are represented as mean (*n* = 3) ± SD; (**b**) cytotoxic effect of doxorubicin, values are represented as mean (*n* = 3) ± SD; (**c**) IC_50_ (µg/mL) of the extract and doxorubicin; (**d**) selectivity index of the extract.

Cytotoxicity was assessed *in vitro* in three cancer cell lines: the prostate (PC-3), breast (MCF-7), and colorectal adenocarcinoma (Caco-2), in comparison with normal Vero cells in response to the treatment with *B. aegyptiaca* methanolic fruit extract. Cytotoxicity increased in a concentration-dependent manner as apparent from the data illustrated in Figure 6. Calculated IC_50_ for *B. aegyptiaca* extract against Vero cells was remarkably high (569.51 µg/mL) compared to MCF-7 (112.31 µg/mL), PC-3 (92.47 µg/mL) and Caco-2 (87.33 µg/mL). This was reflected in the corresponding selectivity index (SI), which reached 5.07, 6.16, and 6.52, respectively. Although the SI induced by the extract treatment against MCF-7 was slightly lower than that recorded for doxorubicin (6.5), the SI’s for the extract against both PC-3 and Caco-2 were higher than those of doxorubicin (1.03 and 1.0, respectively)) PC-3 cells were thus used to further study cell cycle arrest and the expression of apoptosis-related genes induced by the extract treatment. 

### 3.5. Cell Cycle Arrest

**Figure 7:**
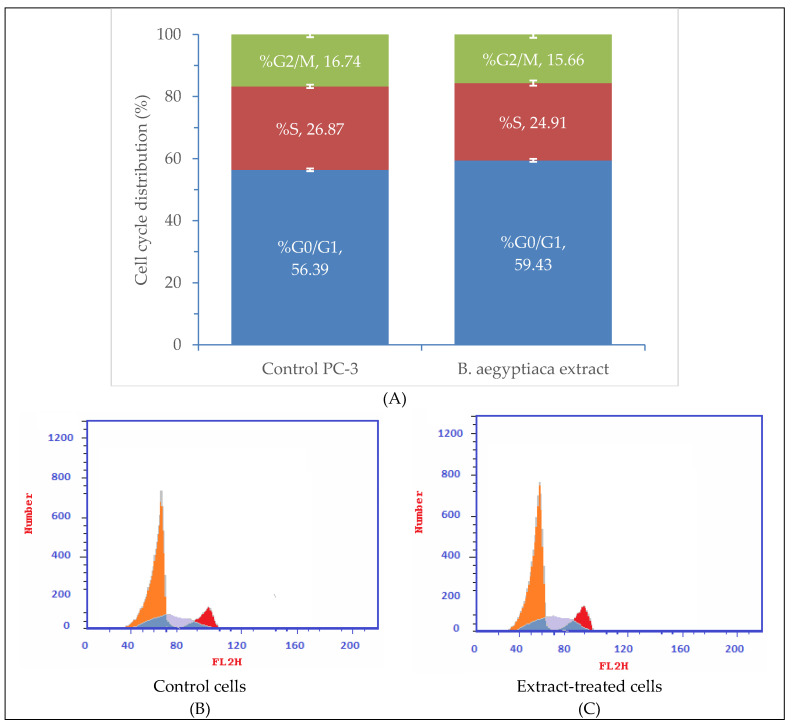
Flow cytometry results of cell cycle distribution of PC-3 cells subjected to *B. aegyptiaca* methanolic fruit extract at 92 µg/mL: (**A**) quantitative cell cycle distribution %. The data presented are the means (*n* = 3) ± SD indicated by the vertical bars. (**B**) DNA content of control cells; (**C**) DNA content of extract-treated cells. Flow cytometry histograms were derived from annexin V-FITC staining assay for PC-3 cells after 24 h incubation in medium alone (**A**) or medium plus *B. aegyptiaca* methanolic fruit extract at 92 µg/mL (**B**).

Analysis of the cell cycle distribution of PC-3 cells treated with *B. aegyptiaca* methanolic fruit extract revealed a slight increase in the percentage of G0/G1 phase (59.43%) compared with the control PC-3 cells (56.39) as illustrated in Figure 7. The percentages of S and G2/M phases, however, were lower in extract-treated cells (24.91 and 15.66%) than those in the control cells (26.87 and 16.74%, respectively). The cell cycle arrest was recorded at G1 phase. 

### 3.6. Apoptosis and Necrosis of Cells

**Figure 8:**
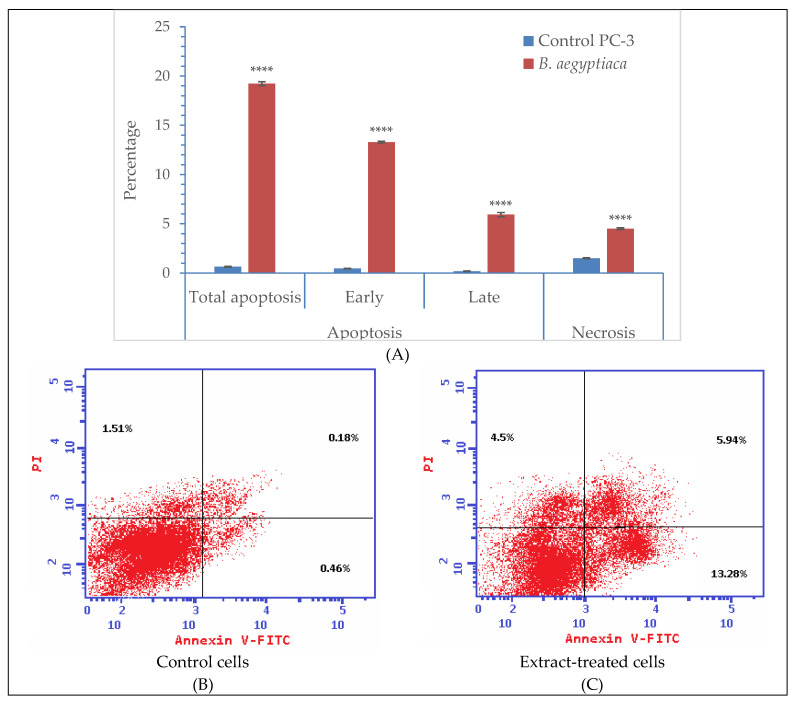
Apoptotic effect of *B. aegyptiaca* methanolic fruit extract at 92 µg/mL on PC-3 cells: (**A**) Proportions of the total, early and late apoptotic cells vs necrotic cells for non-treated and treated PC-3 cells. Statistical differences are indicated by **** at *p* < 0.0001 assessed by independent *t*-test. The data were generated from 3 replicates and presented as means ± SD denoted by the vertical bars. (**B**) flow cytometry dot plots of control cells; (**C**) flow cytometry dot plots of extract-treated cells showing necrotic cells (upper left quadrant), late apoptotic cells (upper right quadrant), viable cells (lower left quadrant), and early apoptotic cells (lower right quadrant).

A potential proapoptotic effect was recorded for *B. aegyptiaca* extract against PC-3 cells as deduced from the analysis results of apoptosis and necrosis in PC-3 cells illustrated in Figure 8. Total apoptosis induced by the extract reached 19.22% including 13.28% early apoptosis and 5.94% late apoptosis in addition to 4.5% necrosis. The control cells, on the other hand, revealed only 0.64% total apoptosis (0.46% early apoptosis and 0.18% late apoptosis) and 1.51% necrosis. 

### 3.7. Expression of Apoptosis-Related Genes

**Figure 9:**
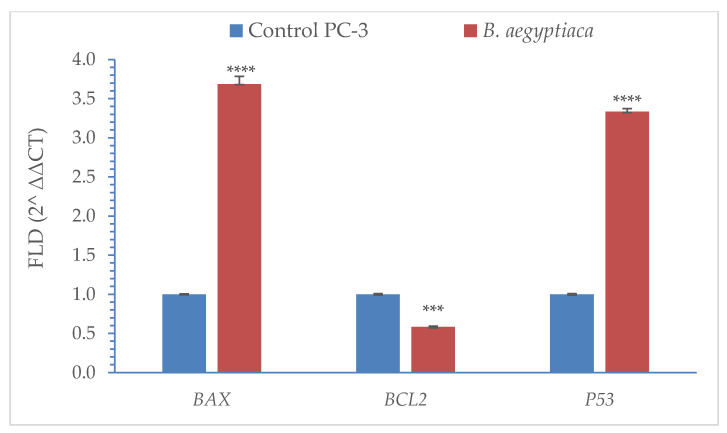
The qRT-PCR results for the expression of apoptosis-related genes in PC-3 cells treated with the *B. aegyptiaca* methanolic fruit extract at 92 µg/mL. Statistical differences are indicated by *** at *p* < 0.001 and **** at *p* < 0.0001 assessed by independent t-test. The data were generated from 3 replicates and presented as means ± SD denoted by the vertical bars.

The expression of *BAX*, *BCL2*, and *P53* genes in PC-3 cells in response to the *B. aegyptiaca* fruit extract at 92 µg/mL was assessed using the qRT-PCR analysis, and the results are illustrated in Figure 9. Relative to the control, the fold change of the proapoptotic genes (*BAX* and *P53*) in extract-treated cells exhibited significant upregulation (3.69 and 3.33, respectively), while the antiapoptotic *BCL2* gene showed significant downregulation (0.58). 

## 4. Discussion

Several studies dealt with the identification of phytochemicals in different parts of *B. aegyptiaca* plant and the investigation of their biological activities inspired by its multipurpose medicinal applications in folk medicine, especially its edible fruits from which it has derived its name: desert dates [3]. In our study, we focused on the fruits, revealing the richness of *B. aegyptiaca* methanolic fruit extract with fatty acids and their esters represented mainly as oleic and palmitic acids (24.12 and 21.56%, respectively). The other components detected belong to phytosterols (*β*-sitosterol), steroid derivatives (ethyl iso-allocholate) and isoflavonoid glycosides (Flavone-4’-OH,5-OH,7-di-O-glucoside). Our results are in line with those previously reported by several authors [3,7,8,16,17] indicating that *B. aegyptiaca* seeds contain approximately 38–57% fats with palmitic and oleic acids estimated to be around 40% of seed oil. Similar results have been reported by other authors such as Al Ashaal [3] and Amadou [1], where palmitic and oleic acids were the prevalent fatty acids in *B. aegyptiaca* seed oil. Other reports showed that the oleic content in the fruits ranged from 33.5% to 69.6% and palmitic from 15.0–17.9% [18]. Phytosterols were also previously reported in fruit extracts such as *β*-Sitosterol and stigmasterol [3]. Phenotypic and genotypic variations have been reported among *B. aegyptiaca* plants collected from different locations, which affected their chemical composition and biological activities [14]. Differences in the phytochemical composition including oil, protein, and some minerals together with the antioxidant activity of fruits collected from different *B. aegyptiaca* trees were reported by several authors, such as Abdel-Farid and El-Sayed [15] and Abdelaziz et al. [4]. In this context, considerable variation in the phytochemical profile of *B. aegyptiaca* methanolic fruit extract is noticeable between the publications. 

When *B. aegyptiaca* fruit extract was studied for its antimicrobial activity, it showed potential antibacterial and antifungal activities. The calculated MIC (62.5 µg/mL) was the same for the three tested bacterial strains (*A. johnsonii*, *S. marcescens*, and *A. tumefaciens*). Meanwhile, it was higher than 1000 µg/mL for the fungal species (*R. solani*, *P. italicum,* and *F. oxysporium*). The human pathogenic bacterium *A. johnsonii* was the most influenced bacterial strain by the extract treatment, while *F. oxysporium* was the most affected fungal species. These results indicate that *B. aegyptiaca* fruit extract is a potent antimicrobial agent according to the categories reported by Kuete [50] classifying substances with MICs below 0.1 mg/mL as potent antimicrobial agents. The antibacterial activity of *B. aegyptiaca* fruit extract has been reported by several previous studies against a number of fungal and bacterial species such as *Candida albicans* and *Staphyloccous aureus* [3,25], *Aspergillus* [3,23,24], *Fusarium* [26,27] and dermatophytes [22]. This effect is related to the bioactive phytochemicals detected in the extract mainly oleic and palmitic acids which were the prevalent components recording 24.12 and 21.56%, respectively. Long-chain saturated fatty acids, including palmitic acid, have been reported to have antibacterial activity and have been used as antimicrobial food additives [28,51]. Methyl palmitate hexadecanoic acid methyl ester showed the highest antimicrobial effect against clinical pathogenic bacteria [52]. Desbois and Smith [53] and Ngamakeue and Chitprasert [54] attributed the high antimicrobial activity against several bacterial strains to the high amount of palmitic acid, possessing the antimicrobial activity. Yff et al. [55] reported palmitic acid as the major antibacterial compound against Gram-positive (*Bacillus subtilis*, *Staphylococcus aureus*) and Gram-negative bacteria (*Escherichia coli*, *Klebsiella pneumoniae*). Similarly, oleic acid derivatives exposed considerable activity against some micro-organisms and were comparable to known antimicrobial agents [56]. 

Our findings revealed a promising potential *in vitro* cytotoxic activity of *B. aegyptiaca* methanolic fruit extract against the breast (MCF-7), prostate (PC-3), and colorectal adenocarcinoma (Caco-2) together with normal Vero cells. The extract induced a high selectivity index (5.07, 6.16 and 6.52) for the three cancer cell lines, respectively. When PC-3 cells were further investigated, they showed cell cycle arrest at G1 phase with a higher G0/G1 phase proportion in extract-treated cells than in the control ones. This effect was mainly proapoptotic as deduced from the high percentage of total apoptosis (19.22%) compared to that in the control cells (0.64%). The proapoptotic effect of the extract was further supported by the upregulation of the proapoptotic genes (*BAX* and *P53*) and the downregulation of the antiapoptotic gene (*BCL2*). In this context, Al-Malki [30] demonstrated cytotoxic effects of *B. aegyptiaca* fruit extract against Hep-2, MCF-7, HL-60, and HCV29T cells with induction of pro-apoptotic effects and modulation of cell cycle phases. Anticancer activities of fatty acids and their esters have been revealed by several previous studies [31,32]. The oil exhibited anticancer activity against lung, liver, and brain human carcinoma cell lines [3]. Bharath et al. [33] recorded in vitro anticancer activity of hexadecanoic acid (palmitic acid) with cell cycle arrest at the G0/G1 phase, which corresponds to our findings. In another study, palmitic acid isolated from *Amphiroa zonata* plant induced cytotoxic activity against human leukemic cells through the inhibition of DNA topoisomerase without affecting normal cells [28,34]. Oleic acid was also found by Jiang et al. [35] to have a proapoptotic effect against tongue squamous cell carcinoma with cell cycle arrest at G0/G1. Sterols and steroidal derivatives present in the extract have also proved a potential anticancer [36]. Ethyl isoallocholate is a steroidal derivative with anticancer activity against A549 lung cancer cells [38]. Flavone 4′-OH,5-OH,7-di-O-glucoside is an isoflavonoid that possesses antioxidant activity [57,58].

These results provide evidence for the promising antimicrobial and cytotoxicity potential of *B. aegyptiaca* methanolic fruit extract against the three cell lines evaluated. However, there has been a limitation that could be considered by future investigations which is the small number of bacterial and fungal strains under study.

## 5. Conclusions

The methanolic extract of *B. aegyptiaca* fruit was found to be rich in fatty acids and their esters with the prevalence of oleic and palmitic acids (24.12 and 21.56%, respectively). The other detected phytochemicals included phytosterols, steroid derivatives, and isoflavonoid glycosides The extract showed high cytotoxic effects against MCF-7, PC-3, and Caco-2 cell lines with high selectivity index against the three cell lines (5.07, 6.16, and 6.52, respectively). The cytotoxic activity was accompanied by high apoptosis induction with upregulation of proapoptotic genes (*BAX* and *P53*) and downregulation of the antiapoptotic *BCL2* gene. *B. aegyptiaca* also induced antimicrobial activity against certain bacterial and fungal species. These promising results represent a base for more detailed studies on the extraction and purification of *B. aegyptiaca* fruits and studying their antiproliferative effect against different other cancer cell lines.

## Figures and Tables

**Table 1:** GC-MS analysis report for the detected phytochemicals in the methanolic fruit extract of *Balanites aegyptiaca*.

No.	Compound	Retention Time (min)	Peak Area %	Chemical Formula	Molecular Weight	CAS Number
1	Octadecanoic acid, ethyl ester (Ethyl Stearate)	4.39	7.58	C_20_H_40_O_2_	312	111-61-5
2	Hexadecanoic acid (Palmitic acid)	18.37	21.56	C_16_H_32_O_2_	256	57-10-3
3	9-octadecenoic acid (Z)- (Oleic acid)	21.03	24.12	C_18_H_34_O_2_	282	112-80-1
4	Octadecanoic acid, 2,3-dihydroxypropyl ester (2,3-Dihydroxypropyl stearate)	21.38	7.12	C_21_H_42_O_4_	358	123-94-4
5	6,9-Octadecadiynoic acid, methyl ester (methyl 6,9-octadecadiynoate)	23.22	12.78	C_19_H_30_O_2_	290	56847-03-1
6	Flavone-4’-OH,5-OH,7-di-O-glucoside	32.29	3.00	C_27_H_30_O_15_	594	NA *
7	Stigmast-5-en-3-ol, (3 beta)- (β-sitosterol)	34.84	15.57	C_29_H_50_O	414	83-46-5
8	Ethyl iso-allocholate (Ethyl cholate)	36.42	8.27	C_26_H_44_O_5_	436	NA

* NA = Not applicable.

## Data Availability

The data that support the findings of this study are available on request from the corresponding author (O.H.M.I.). The three tested cancer cell lines (MCF-7, PC-3, and Caco-2) are commercially available.
